# Two decades of progress in glioma methylation research: the rise of temozolomide resistance and immunotherapy insights

**DOI:** 10.3389/fnins.2024.1440756

**Published:** 2024-09-02

**Authors:** Xianhao Huo, Haoyuan Li, Yixiang Xing, Wenqing Liu, Pengfei Chen, Fang Du, Lijuan Song, Zhenhua Yu, Xiangmei Cao, Jihui Tian

**Affiliations:** ^1^Department of Neurosurgery, General Hospital of Ningxia Medical University, Yinchuan, China; ^2^Ningxia Key Laboratory of Cerebrocranial Disease, Ningxia Medical University, Yinchuan, China; ^3^Clinical Medical College, Ningxia Medical University, Yinchuan, China; ^4^School of Information Engineering, Ningxia University, Yinchuan, China; ^5^Collaborative Innovation Center for Ningxia Big Data and Artificial Intelligence Co-founded by Ningxia Municipality and Ministry of Education, Ningxia University, Yinchuan, China; ^6^Basic Medical School, Ningxia Medical University, Yinchuan, China

**Keywords:** glioma methylation, *MGMT* promoter methylation, temozolomide, immunotherapy, bibliometric analysis

## Abstract

**Aims:**

This study aims to systematically analyze the global trends in glioma methylation research using bibliometric methodologies. We focus on identifying the scholarly trajectory and key research interests, and we utilize these insights to predict future research directions within the epigenetic context of glioma.

**Methods:**

We performed a comprehensive literature search of the Web of Science Core Collection (WoSCC) to identify articles related to glioma methylation published from January 1, 2004, to December 31, 2023. The analysis included full-text publications in the English language and excluded non-research publications. Analysis and visualization were performed using GraphPad Prism, CiteSpace, and VOSviewer software.

**Results:**

The search identified 3,744 publications within the WoSCC database, including 3,124 original research articles and 620 review articles. The research output gradually increased from 2004 to 2007, followed by a significant increase after 2008, which peaked in 2022. A minor decline in publication output was noted during 2020–2021, potentially linked to the coronavirus disease 2019 pandemic. The United States and China were the leading contributors, collectively accounting for 57.85% of the total research output. The Helmholtz Association of Germany, the German Cancer Research Center (DKFZ), and the Ruprecht Karls University of Heidelberg were the most productive institutions. The Journal of Neuro-Oncology led in terms of publication volume, while Neuro-Oncology had the highest Impact Factor. The analysis of publishing authors revealed Michael Weller as the most prolific contributor. The co-citation network analysis identified David N. Louis's article as the most frequently cited. The keyword analysis revealed “temozolomide,” “expression,” “survival,” and “DNA methylation” as the most prominent keywords, while “heterogeneity,” “overall survival,” and “tumor microenvironment” showed the strongest citation bursts.

**Conclusions:**

The findings of this study illustrate the increasing scholarly interest in glioma methylation, with a notable increase in research output over the past two decades. This study provides a comprehensive overview of the research landscape, highlighting the importance of temozolomide, DNA methylation, and the tumor microenvironment in glioma research. Despite its limitations, this study offers valuable insights into the current research trends and potential future directions, particularly in the realm of immunotherapy and epigenetic editing techniques.

## 1 Introduction

Glioma, a type of central nervous system neoplasm, is the most prevalent form of malignant intracranial neoplasm, accounting for ~40%−50% of all such tumors (Louis et al., 2021). Gliomas are distinguished by their high invasiveness and malignancy, which contribute to substantial morbidity and mortality. Despite the utilization of a multimodal therapeutic regimen encompassing surgical intervention, radiotherapy, and chemotherapy, the long-term prognosis of glioma is often unfavorable. Glioma management presents a formidable clinical challenge due to its high degree of heterogeneity and resistance to conventional treatments. As such, glioma management is an important topic within the realm of medical research.

Recent scientific inquiry has revealed the pivotal role of epigenetic regulation in the etiology, progression, and therapeutic intervention of malignant neoplasms such as glioma (Capper et al., 2018b; Romani et al., 2018; Pan et al., 2021; Ozair et al., 2023). Methylation modification, a fundamental mechanism in epigenetic regulation, encompasses several components, including DNA methylation, RNA methylation, and histone methylation, each contributing to the intricate biological behaviors exhibited by cancer cells. A growing body of evidence in glioma research has underscored the influence of methylation modification in the malignant phenotype, stemness (Du et al., 2021; Mao et al., 2022; Sharma et al., 2023), and chemosensitivity to agents such as temozolomide (Wick et al., 2014; Zhou et al., 2015; Zhang and Zhu, 2019; Zapanta et al., 2024), as well as in the responsiveness to radiotherapy.

The present study used bibliometric methodologies to systematically analyze the existing literature pertaining to glioma methylation, as indexed within the Web of Science Core Collection (WoSCC) database, spanning a period of two decades. We produced a comprehensive knowledge map to illustrate the trajectory of research in this field and discern the focal points of scholarly interest. The overarching aims of this study are to provide a robust theoretical framework and to offer prescriptive guidance for future glioma research, specifically from an epigenetic vantage point. This approach is anticipated to support the genesis and refinement of innovative therapeutic modalities targeted at this intractable tumor.

## 2 Materials and methods

Compared with other databases, the WoSCC database excels in the accuracy of document type annotation, making it the optimal choice for literature analysis (Jiang et al., 2023; Mao et al., 2023). Therefore, we performed our literature search within the WoSCC database. On March 2, 2024, we searched the WoSCC database to identify all publications related to the study of glioma methylation published between January 1, 2004, and December 31, 2023, using the following search formula: (((((((((((((TS=(glioma)) OR TS=(Gliomas)) OR TS=(Glial Cell Tumors)) OR TS=(Glial Cell Tumor)) OR TS=(Tumor, Glial Cell)) OR TS=(Tumors, Glial Cell)) OR TS=(Mixed Glioma)) OR TS=(Glioma, Mixed)) OR TS=(Gliomas, Mixed)) OR TS=(Malignant Glioma)) OR TS=(Glioma, Malignant)) OR TS=(Gliomas, Malignant)) OR TS=(Malignant Gliomas)) AND TS=(methylation) (Supplementary material).

**Figure 1:**
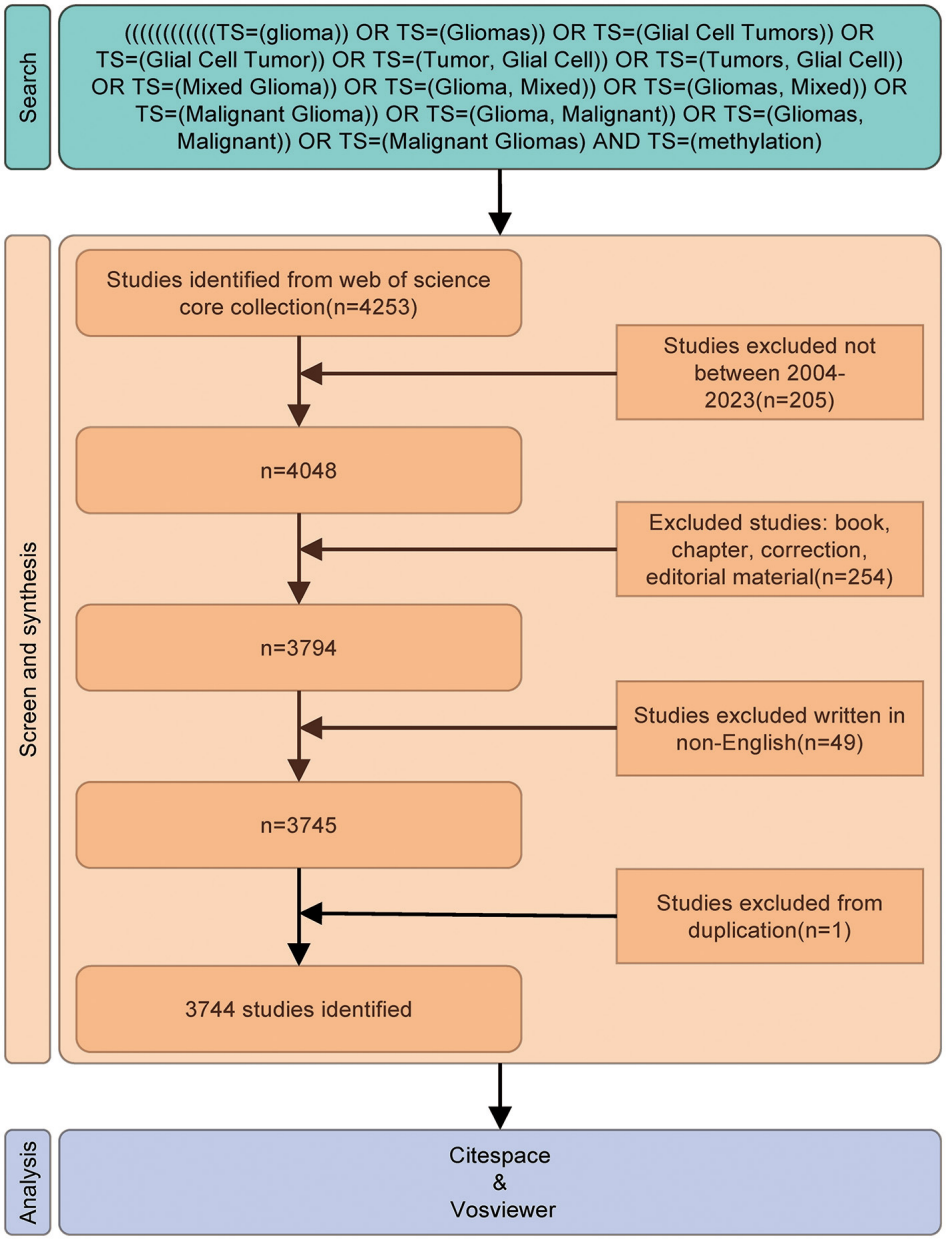
Flowchart of the study. The different colors represent the various stages of the study; green represents the search phase with the key terms identified, orange represents screening, and blue represents the analysis and visualization.

Published studies were selected according to the following inclusion criteria: (1) full-text publications on the study of glioma methylation; (2) articles and review manuscripts written in the English language; and (3) documents published between January 1, 2004, and December 31, 2023. The exclusion criteria included (1) topics unrelated to the study of glioma methylation and (2) non-research publications, such as conference abstracts, news articles, and brief reports. The full-text versions of the selected papers were exported. The flow diagram of the study is shown in Figure 1.

VOSviewer (van Eck and Waltman, 2010) is a free JAVA-based software designed to analyze and graphically display large bibliometric datasets. To visualize research outcomes in a specific field through co-citation network mapping, professor Chen Chaomei developed CiteSpace software (Chen and Leydesdorff, 2014). CiteSpace uses an experimental framework to explore new concepts and evaluate current methodologies, enabling users to gain a deeper understanding of knowledge domains, research frontlines, and trends, and to forecast potential future research directions. Centrality is an important indicator in Citespace, the larger the centrality value, the more important it is in the network.

In the analysis, nodes in the graph represent analyzed objects, such as countries, institutions, journals, authors, and keywords. The links between nodes indicate relationships, with line thickness reflecting the strength of association. Centrality represents the degree of importance of a node which quantified by higher values signifies greater importance. Cluster analysis groups keywords, differentiated by color, with smaller cluster labels indicating a larger number of keywords. Burst detection identifies keywords with sudden increases in frequency, marked by red intervals for the years of surge.

In this study, VOSviewer (version 1.6.18) and CiteSpace (version 6.2.4R, 64-bit, advanced edition) were used to analyze the data and visualize the scientific knowledge map. Moreover, GraphPad Prism (version 8.0.2) was used to analyze and plot the annual publication trends and national distribution ratios.

## 3 Results

The findings revealed that between January 1, 2004, and December 31, 2023, the WoSCC database had compiled an extensive archive of publications on glioma methylation amounting to 3,744 documents. This included 3,124 original research articles (84.96%) and 620 review articles (15.04%). Global collaborative research efforts were reflected in these publications, with contributions from institutions in 86 distinct countries and regions. The research community was broad, encompassing 3,508 institutions and 19,444 individual authors.

### 3.1 Analysis of article publishing trends

**Figure 2:**
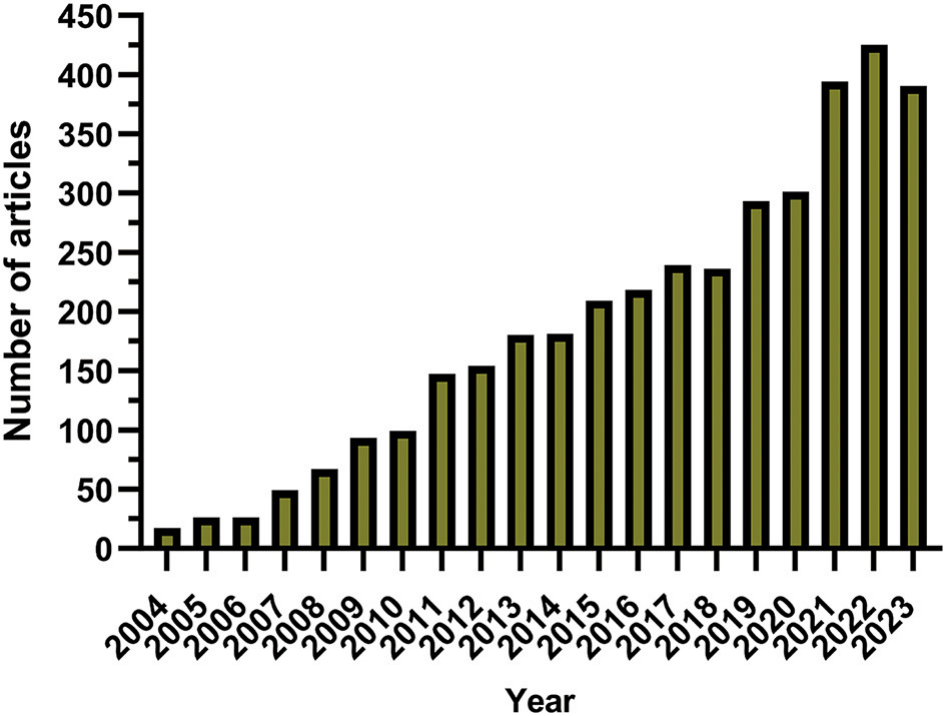
Annual publications related to glioma and methylation from January 1, 2004, to December 31, 2023.

The annual scholarly publication output was characterized by an upward trajectory from 2004 (Figure 2). The initial phase spanning from 2004 to 2007 was characterized by a gradual increase in the dissemination of academic papers, but the annual count never surpassed 50 publications. After 2008, there was an increase in the publication volume, which peaked in 2022. A transient downturn in publication output was observed during 2020–2021, a deviation potentially linked to the worldwide outbreak of the coronavirus disease 2019 pandemic.

### 3.2 Analysis of the article origins by country and institution

**Figure 3:**
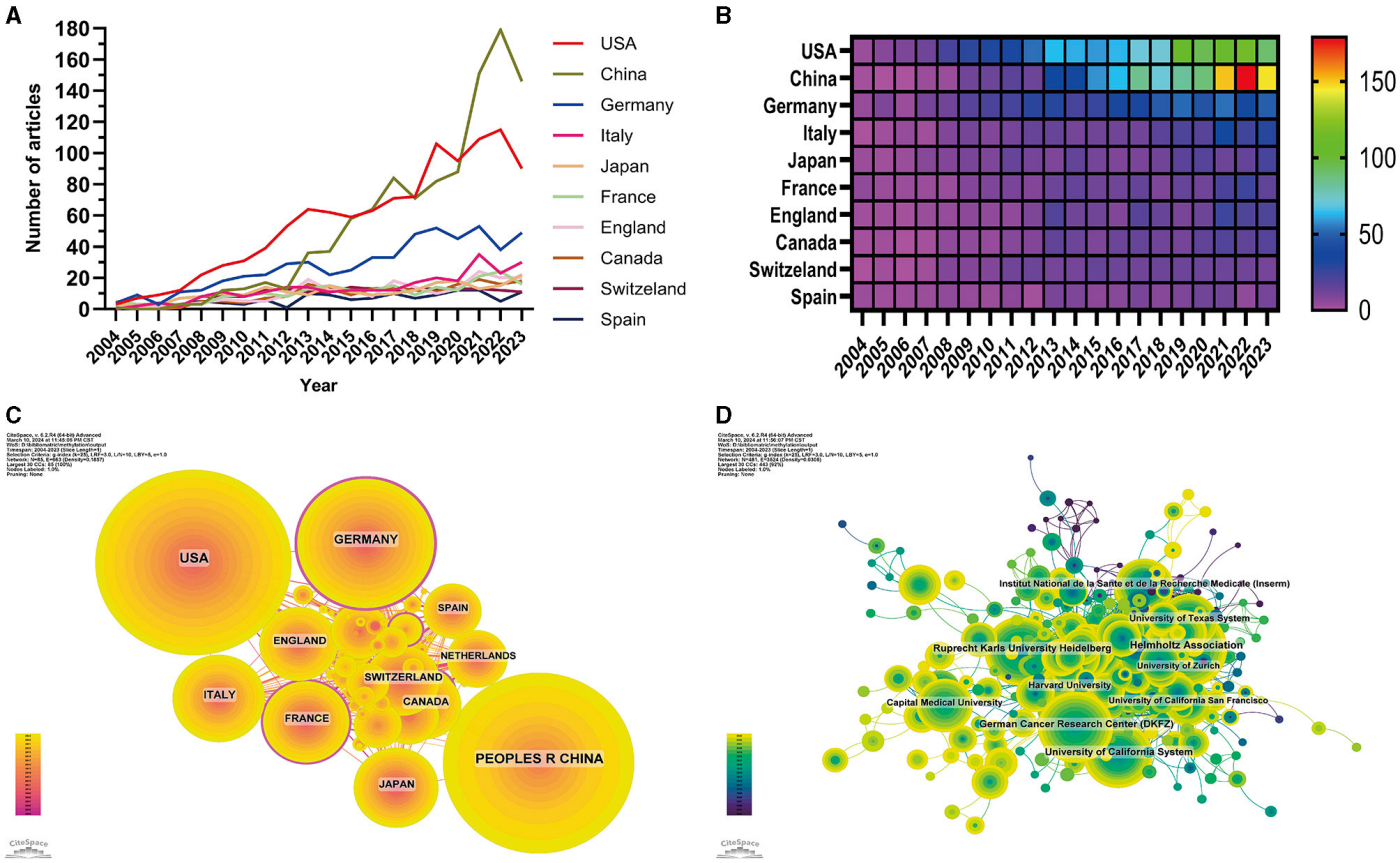
Visualization of the country and institution analysis. Line chart **(A)** and heatmap **(B)** of annual publications from the top 10 contributing countries. Collaboration visualization among countries **(C)** and institutions **(D)**. The node size represents the number of published articles. The connecting lines represent the strengths of the relationships.

A comprehensive evaluation identified 86 countries and regions actively engaged in the study of glioma methylation. The annual research output of the top 10 nations over the past 10 years is articulated in Figures 3A, B. An in-depth evaluation of the research productivity of these leading countries is presented in Table 1, which shows their publication volumes and respective shares. The United States, China, Germany, Italy, and Japan emerged as the five leading countries. The aggregate research output of the United States and China accounted for 57.85% of all publications, outstripping that of the other countries by a considerable margin. A detailed analysis of the most productive institutions highlighted the dominance of the Helmholtz Association of Germany, with 253 publications, followed by the German Cancer Research Center (DKFZ) and the Ruprecht Karls University of Heidelberg with 232 and 196 publications, respectively (Table 1).

**Table 1:** Top 10 countries (left) and institutions (right) involved in glioma methylation research.

**Rank**	**Country**	**Article counts**	**Centrality**	**Percentage (%)**	**Rank**	**Institution (Country)**	**Study counts**	**Citations**
1	USA	1,108	0.10	29.59	1	Helmholtz Association (Germany)	253	24,365
2	China	1,058	0.02	28.26	2	German Cancer Research Center (DKFZ) (Germany)	232	23,588
3	Germany	558	0.26	14.90	3	Ruprecht Karls University Heidelberg (Germany)	196	27,033
4	Italy	264	0.07	7.05	4	University of California System (USA)	192	32,355
5	Japan	218	0.03	5.82	5	Harvard University (USA)	136	25,097
6	France	215	0.15	5.74	6	Institut National de la Sante et de la Recherche Medicale (Inserm) (France)	132	14,879
7	England	207	0.05	5.53	7	Capital Medical University (China)	127	3,323
8	Canada	190	0.08	5.07	8	University of Texas System (USA)	126	22,368
9	Switzerland	189	0.03	5.05	9	University of Zurich (Switzerland)	120	18,937
10	Netherlands	127	0.07	3.39	10	University of California San Francisco (USA)	112	24,519

In terms of the collaborative research patterns among the nations and institutions, we observed a robust and intimate research partnership between the United States and China (Figure 3C). In terms of institutional collaborations, we identified a preference among the majority of research entities for forming alliances with domestic counterparts (Figure 3D), while cross-national collaborations were less frequent.

### 3.3 Analysis of publishing and co-citing journals

**Figure 4:**
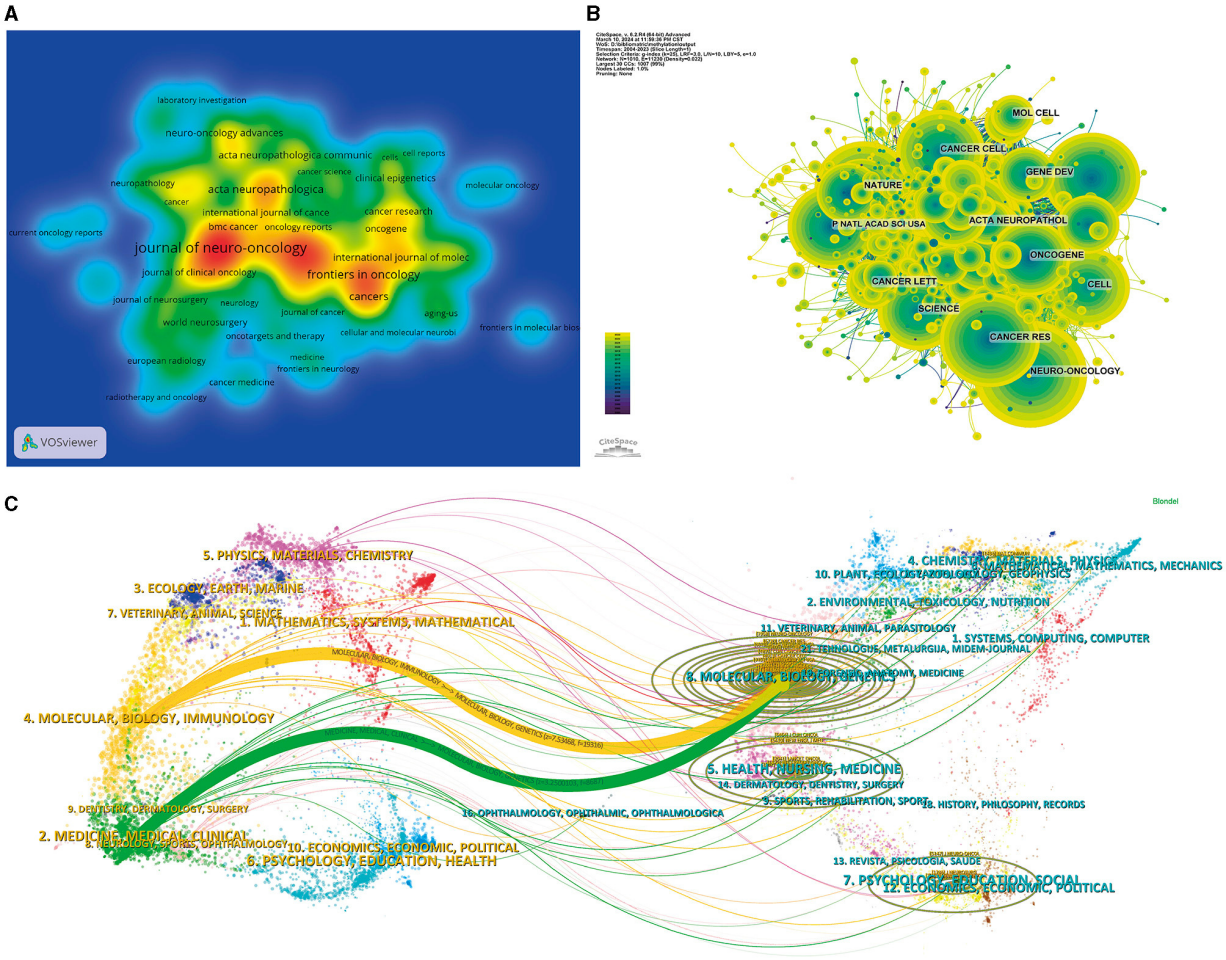
Visualization of the journal analysis. **(A)** Density graph of journal publications. The red parts represent more publications than the blue parts. **(B)** Network visualization of the co-cited journals. The node size represents the number of published articles. The connecting lines represent the strengths of the relationships. **(C)** Dual-map overlays of the cited journals and the citing journals.

Table 2 shows the leading journals by publication volume, and the corresponding heatmap of their publications is depicted in Figure 4A. Prominent among the journals was the Journal of Neuro-Oncology, which claimed the leading position with 191 publications (5.10% of the total analyzed). This journal was closely followed by Neuro-Oncology with 137 publications (3.66%), and Frontiers in Oncology with 99 publications (4.7%). Notably, within this elite group of journals, Neuro-Oncology had the highest Impact Factor (15.9), and all were recognized within the prestigious Q1 or Q2 ranking categories.

**Table 2:** Top 10 journals (left) and co-cited journals (right) involved in glioma methylation research.

**Rank**	**Journal**	**Article counts**	**IF (2022)**	**Quartile in category (2022)**	**Rank**	**Cited journal**	**Co-Citation**	**IF (2022)**	**Quartile in category (2022)**
1	Journal Of Neuro-Oncology	191	3.9	Q2	1	Neuro-Oncology	2,535	15.9	Q1
2	Neuro-Oncology	137	15.9	Q1	2	New England Journal Of Medicine	2,364	158.5	Q1
3	Frontiers In Oncology	99	4.7	Q2	3	Cancer Research	2,269	11.2	Q1
4	Cancers	85	5.2	Q2	4	Clinical Cancer Research	2,046	11.5	Q1
5	Acta Neuropathologica	70	12.7	Q1	5	Nature	2,025	64.8	Q1
6	Plos One	68	3.7	Q2	6	Journal Of Neuro-Oncology	2,010	3.9	Q2
7	Scientific Reports	52	4.6	Q2	7	Acta Neuropathologica	1,972	12.7	Q1
8	Clinical Cancer Research	51	11.5	Q1	8	Journal Of Clinical Oncology	1,943	45.4	Q1
9	International Journal Of Molecular Sciences	45	5.6	Q1	9	Proceedings Of The National Academy Of Sciences Of The United States Of America	1,671	11.1	Q1
10	Acta Neuropathologica Communications	43	7.1	Q1	10	Cancer Cell	1,627	50.3	Q1

The scholarly influence of a journal is often measured by the citation frequency of its publications. Figure 4B, Table 2 show that Neuro-Oncology had the highest co-citation frequency of 2,535, followed by New England Journal of Medicine with 2,364 citations and Cancer Research with 2,269 citations. Among the top 10 journals by citation frequency, New England Journal of Medicine had the second highest citation frequency and the highest Impact Factor (158.5). All journals within this citation frequency hierarchy areplaced within the esteemed Q1 or Q2 quadrant.

The thematic distribution within the field of glioma methylation research is illustrated in Figure 4C. The colored pathways symbolize citation links, with the citing journals on the left and the cited journals on the right. The visual analysis shows two primary citation trajectories. First, research from the molecular/biology/immunology domain was predominantly referenced by research within the molecular/biology/genetics domain. Second, studies within the medicine/medical/clinical domain were also predominantly cited by research from the molecular/biology/genetics domain.

### 3.4 Analysis of publishing authors

**Figure 5:**
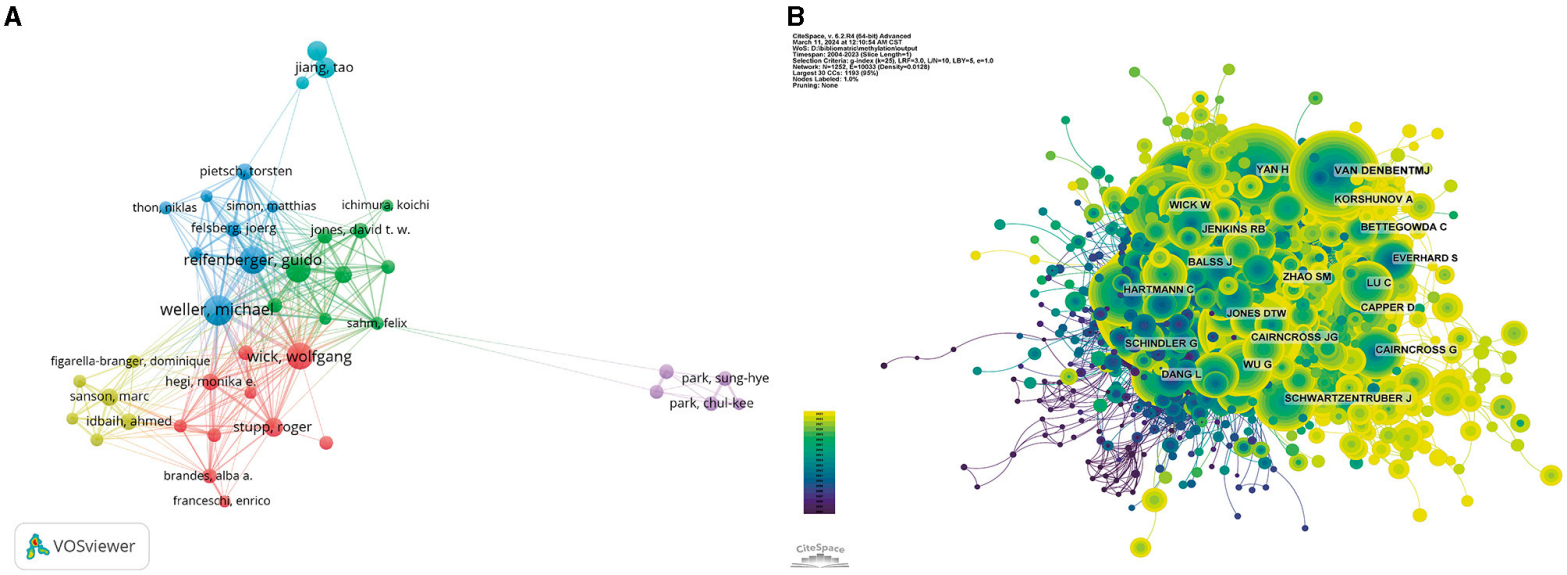
Visualization of the author analysis. Network visualization of the author collaborations **(A)** and co-cited authors **(B)**. The different colors represent different clusters. Larger nodes represent more articles.

In tribute to the scholars whose contributions have been pivotal to the field of glioma methylation research, we meticulously identified the leading authors, as distinguished by their publication records (Table 3). At the time of writing, these esteemed authors had collectively authored 482 publications, constituting 12.88% of the entire body of research on this topic. Michael Weller led with an impressive 79 publications, succeeded by Guido Reifenberger with 68 publications, Wolfgang Wick with 65, Andreas von Deimling with 57, and Tao Jiang with 43. A geographical survey of these academic leaders identified their origins. There were four from Germany, three from Switzerland, two from China, and one from France. CiteSpace was used to produce a visual representation of the collaborative relationships among these authors (Figure 5A).

**Table 3:** Top 10 authors (left) and co-cited authors (right) involved in glioma methylation research.

**Rank**	**Author**	**Counts**	**Location**	**Rank**	**Co-cited author**	**Citations**
1	Michael Weller	79	Switzerland	1	David N. Louis	1,418
2	Guido Reifenberger	68	Germany	2	Roger Stupp	1,310
3	Wolfgang Wick	65	Germany	3	Monika E. Hegi	1,244
4	Andreas Von Deimling	57	Germany	4	Michael Weller	762
5	Tao Jiang	43	China	5	Manel Esteller	693
6	Wei Zhang	41	China	6	Hai Yan	613
7	Roger Stupp	37	Switzerland	7	Quinn T Ostrom	545
8	David Capper	32	Germany	8	Martin J van den Bent	540
9	Marc Sanson	31	France	9	Wolfgang Wick	538
10	Monika E. Hegi	29	Switzerland	10	Patrick Y. Wen	473

Figure 5B highlights the most co-cited authors based on their citation frequency above 50 citations. Within the co-citation network, the size of each node is proportional to the citation frequency, with the most prominent nodes representing the authors who have been most frequently co-cited, including David N Louis with 1,418 citations, Roger Stupp with 1,310 citations, and Monika E Hegi with 1,244 citations.

### 3.5 Analysis of references

**Figure 6:**
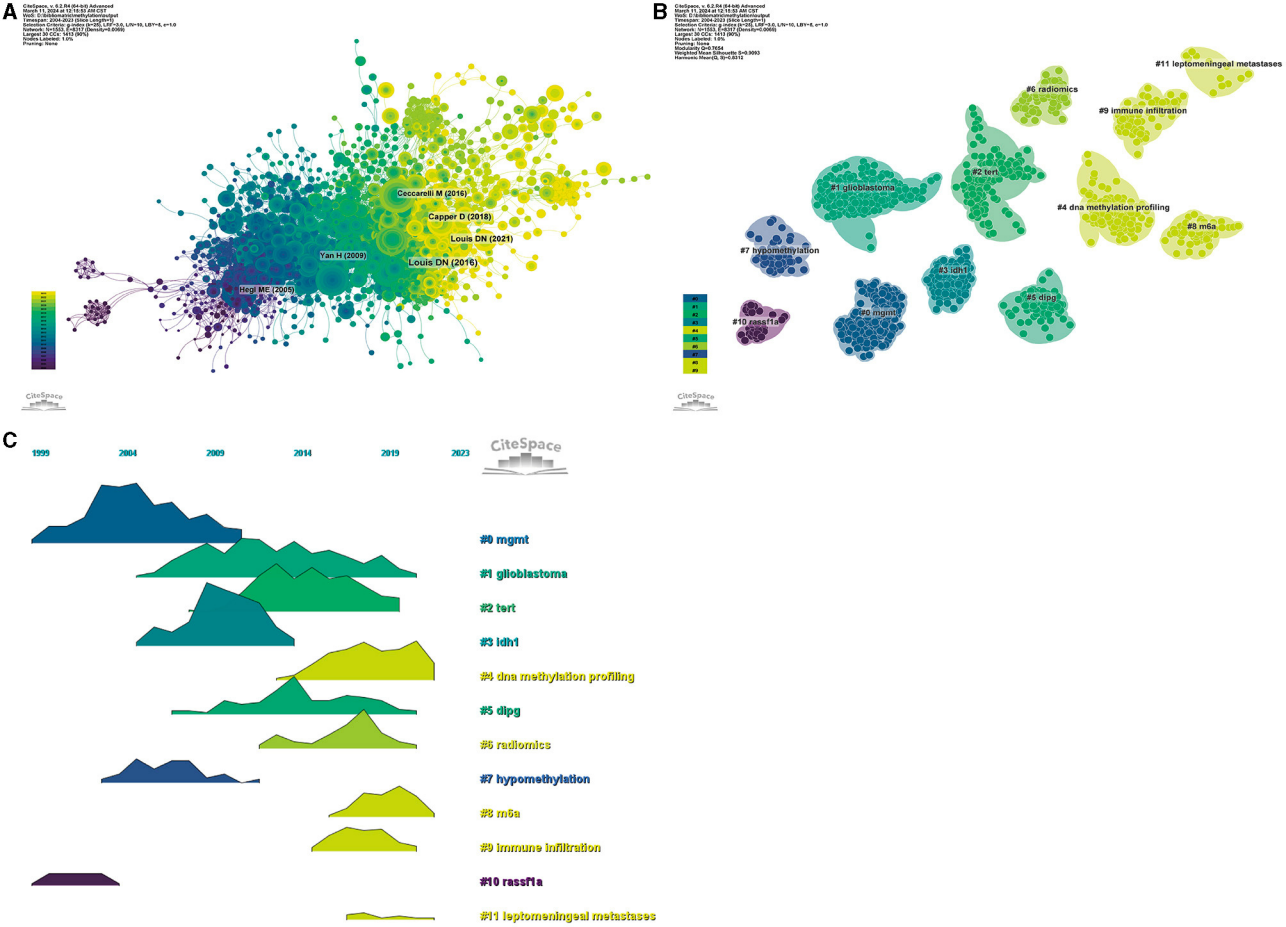
Visualization of the co-cited reference analysis. Network relationship **(A)**, clustering according to publication year **(B)**, and volcano plot of co-cited references **(C)**.

The co-citation network analysis from 2004 to 2023 produced a comprehensive map of 1,553 nodes interconnected by 8,317 relationships (Figure 6A). Of the most highly co-cited publications presented in Table 3, the dominant position was occupied by David N. Louis's seminal article, “The 2016 World Health Organization Classification of Tumors of the Central Nervous System: A Summary,” published in Acta Neuropathologica, which accumulated a substantial count of 487 citations. A close second was the groundbreaking study by David Capper, titled “DNA methylation-based classification of central nervous system tumors,” published in Nature, which garnered 284 citations (Table 4).

**Table 4:** Top 10 co-cited references related to glioma methylation research.

**Rank**	**Title**	**IF (2022)**	**Author**	**PMID**	**Year**	**Citations**
1	The 2016 world health organization classification of tumors of the central nervous system: a summary (Louis et al., 2016)	Acta Neuropathologica (IF=12.7)	David N. Louis	27157931	2016	487
2	DNA methylation-based classification of central nervous system tumors (Capper et al., 2018a)	Nature (IF=64.8)	David Capper	29539639	2018	284
3	The 2021 WHO classification of tumors of the central nervous system: a summary (Louis et al., 2021)	Neuro-Oncology (IF=15.9)	David N. Louis	34185076	2021	272
4	*IDH1* and *IDH2* mutations in gliomas (Yan et al., 2009)	New England Journal of Medicine (IF=158.5)	Hai Yan	19228619	2009	183
5	Molecular profiling reveals biologically discrete subsets and pathways of progression in diffuse glioma (Ceccarelli et al., 2016)	Cell (IF=64.5)	Michele Ceccarelli	26824661	2016	182
6	*MGMT* gene silencing and benefit from temozolomide in glioblastoma (Hegi et al., 2005)	New England Journal of Medicine (IF=158.5)	Monika E. Hegi	15758010	2005	181
7	Comprehensive, integrative genomic analysis of diffuse lower-grade gliomas (Brat et al., 2015)	New England Journal of Medicine (IF=158.5)	Daniel J. Brat	26061751	2015	174
8	Effects of radiotherapy with concomitant and adjuvant temozolomide vs. radiotherapy alone on survival in glioblastoma in a randomized phase III study: 5-year analysis of the EORTC-NCIC trial (Stupp et al., 2009)	Lancet Oncology (IF=51.1)	Roger Stupp	19269895	2009	164
9	Integrated genomic analysis identifies clinically relevant subtypes of glioblastoma characterized by abnormalities in *PDGFRA, IDH1, EGFR*, and *NF1* (Verhaak et al., 2010)	Cancer Cell (IF=50.3)	Roel G. W. Verhaak	20129251	2010	160
10	The somatic genomic landscape of glioblastoma (Brennan et al., 2013)	Cell (IF=64.5)	Cameron W. Brennan	24120142	2013	149

Utilizing a methodological approach that integrates co-citation clustering and temporal analysis (Figures 6A, B), we discerned the temporal trajectory of research focal points. The early research periods were marked by an emphasis on mgmt (cluster 0) and rassf1a (cluster 10). Subsequent periods witnessed a shift toward Idh1 (cluster 3) and hypomethylation (cluster 7). In more recent years, the contemporary research landscape has been dominated by other pivotal topics, including glioblastoma (cluster 1), *TERT* (cluster 2), DNA methylation profiling (cluster 4), dipg (cluster 5), radiomics (cluster 6), m6A (cluster 8), immune infiltration (cluster 9), and leptomeningeal metastases (cluster 11).

### 3.6 Analysis of keywords

**Figure 7:**
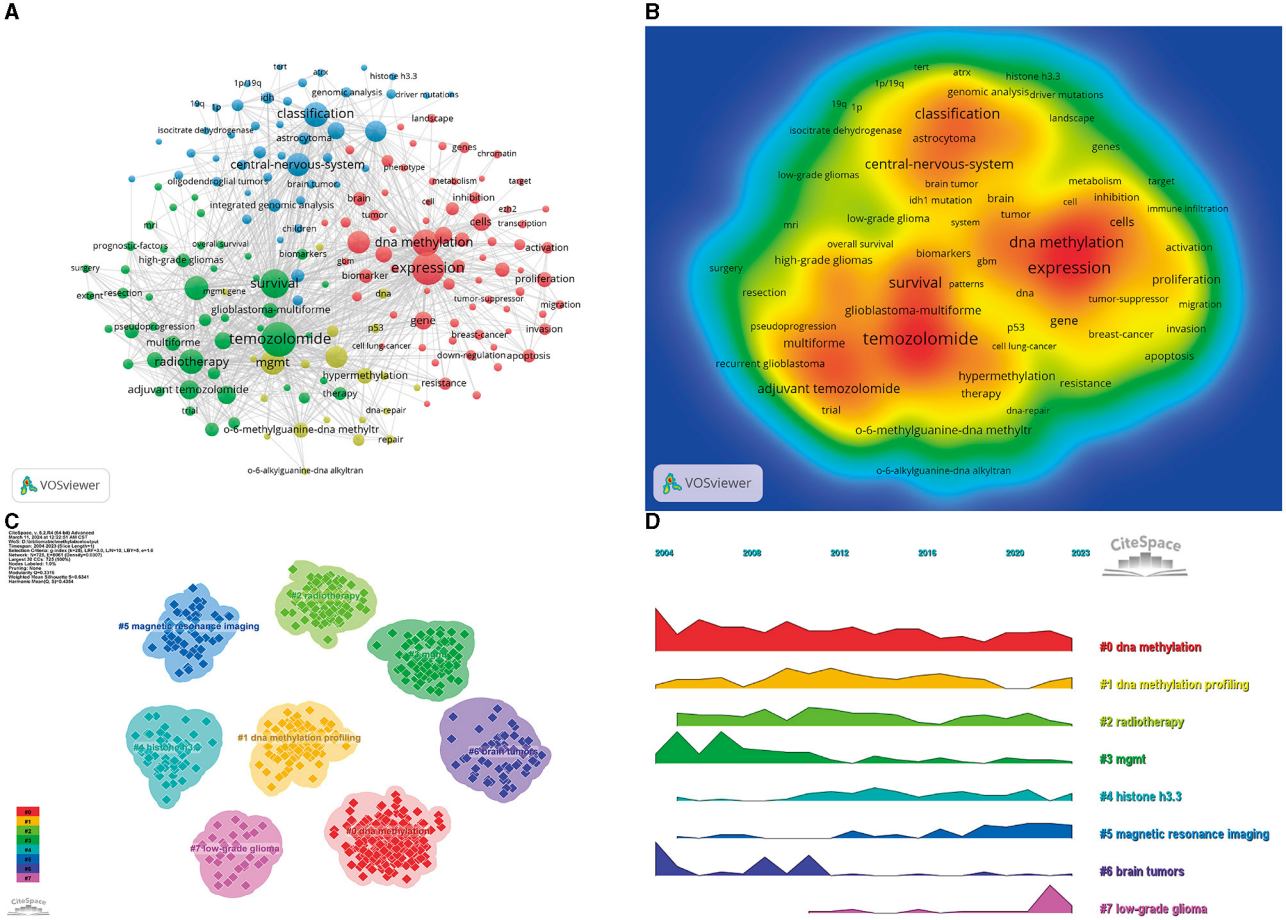
Visualization of the keyword analysis. Network of high-frequency keywords **(A)**, density graph **(B)**, clustering visualization **(C)**, and keyword clustering volcano map **(D)**.

Keyword analysis is a pivotal methodology that can be used to capture the landscape and evolutionary trajectory of a specific research domain. Utilizing the co-occurrence dataset of keywords from VOSviewer, the most commonly featured terms were “temozolomide” (869 occurrences), “expression” (767 occurrences), “survival” (600 occurrences), and “DNA methylation” (514 occurrences) (Table 5, Figures 7A, B). Following the exclusion of less frequently utilized keywords, a network of 185 keywords, each appearing no fewer than 34 times, was assembled and stratified into four discrete clusters. The initial cluster (red) incorporates an array of keywords, including, but not limited to, “DNA methylation,” “prognosis,” “mechanisms,” “differentiation,” and associated terms. The subsequent cluster (green) encompasses 47 keywords, predominantly related to “temozolomide,” “radiotherapy,” and “mgmt promoter methylation.” The third cluster (blue) comprises 46 keywords, with a focus on “classification,” “central nervous system,” “integrated genomic analysis,” and related terminologies. The fourth cluster (yellow) encompasses 21 keywords, including “association,” “DNA repair gene,” “hypermethylation,” and other pertinent terms. The temporal shifts in the research focal points are depicted as a volcano plot, which was generated using CiteSpace software. The volcano plot intuitively portrays the progression of the research hotspots over time (Figures 7C, D).

**Table 5:** Top 20 keywords related to glioma methylation research.

**Rank**	**Keyword**	**Counts**	**Rank**	**Keyword**	**Counts**
1	Temozolomide	869	11	Promoter methylation	352
2	Expression	767	12	Mutations	332
3	Survival	600	13	Adjuvant temozolomide	280
4	DNA methylation	514	14	Malignant glioma	252
5	Classification	454	15	Gene-expression	221
6	*MGMT*	449	16	*IDH1*	218
7	Radiotherapy	443	17	Chemotherapy	214
8	*MGMT* Promoter methylation	422	18	Progression	199
9	Central-nervous-system	394	19	Newly-diagnosed glioblastoma	196
10	Prognosis	370	20	Hypermethylation	194

### 3.7 Analysis of references and keywords with the strongest citation bursts

Utilizing CiteSpace software, we curated a list of the 50 most influential and highly cited publications in glioma methylation research. Among them was the influential publication by David N. Louis published in Acta Neuropathologica, which presents a comprehensive overview of the “The 2016 World Health Organization Classification of Tumors of the Central Nervous System: A Summary”, achieving a citation frequency of 175.16. The temporal distribution of these publications spanning from 2004 to 2023 underscores their sustained relevance and frequent citation over two decades.

**Figure 8:**
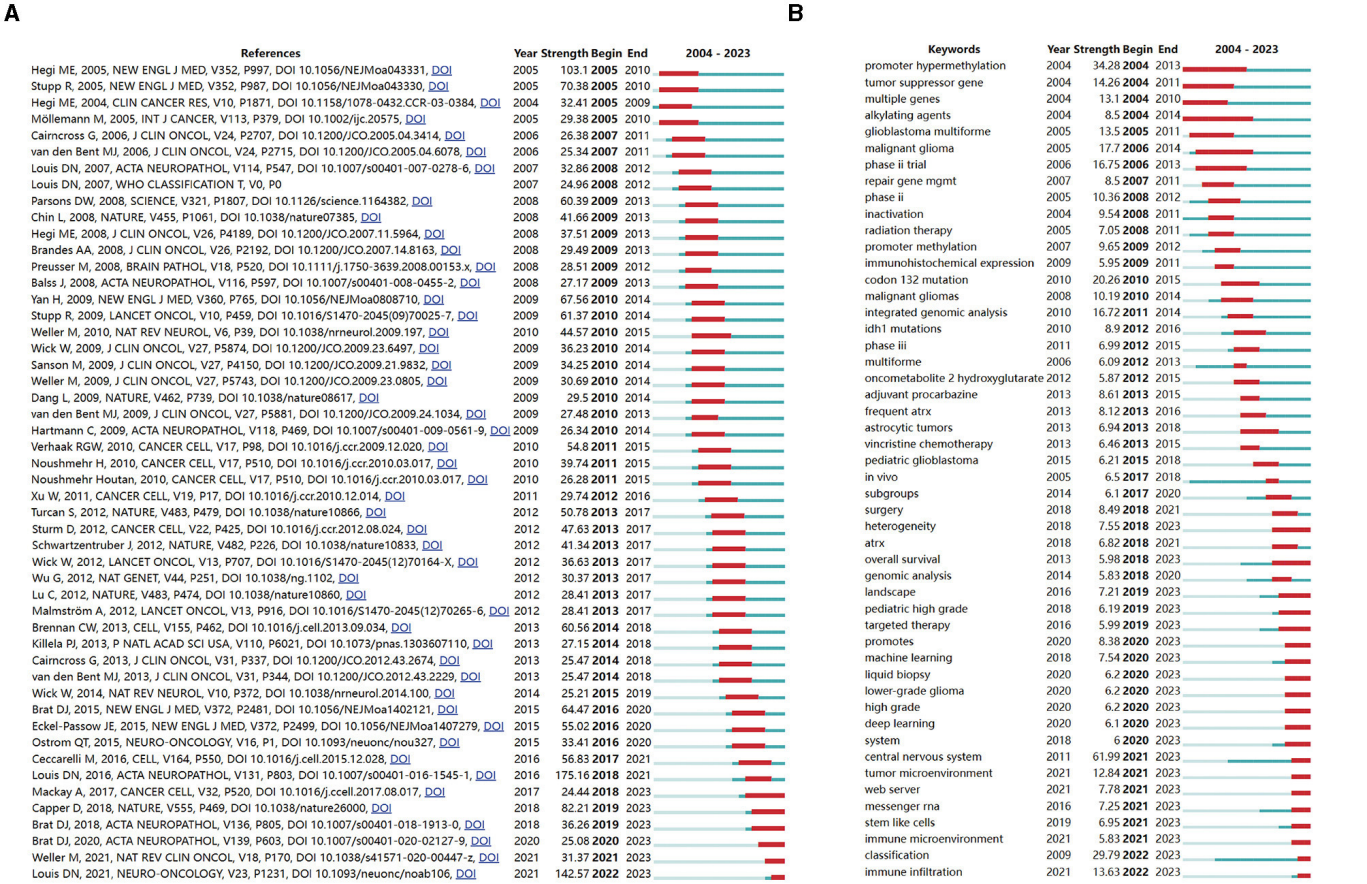
Analysis of the top 50 references **(A)** and keywords **(B)** with the strongest citation bursts. The blue line indicates the keyword occurrence and its duration. The red line indicates the burst time and duration.

Among the 50 publications, six articles remained in an active phase of citation intensity at the time of writing (Figure 8A), which signifies the ongoing and future prominence of glioma methylation research.

We evaluated the most salient keywords, focusing on the 50 keywords with the highest burst strength among a total of 867 keywords (Figure 8B). The keywords that had garnered persistent interest as of 2023 included “heterogeneity,” “overall survival,” “landscape,” “pediatric high grade,” “targeted therapy,” “promotes,” “machine learning,” “liquid biopsy,” “lower-grade glioma,” “high grade,” “deep learning,” “system,” “central nervous system,” “tumor microenvironment,” “web server,” “messenger RNA,” “stem-like cells,” “immune microenvironment,” “classification,” and “immune infiltration.” These keywords not only encapsulate the recent focal points of research efforts but also signal the future trajectories of glioma methylation research.

## 4 Discussion

This study is the first to use bibliometric methodologies to systematically evaluate glioma methylation research. This study constructed a dataset comprising 3,744 publications indexed within the WoSCC database from 2004 to 2023. We performed a detailed visualization analysis using CiteSpace and VOSviewer software to evaluate the contributing authors, affiliated institutions, publishing journals, and pivotal keywords. The synthesis of these data produced a knowledge network map, which shows the landscape of glioma methylation research over the past two decades and offers an insightful perspective into the current research paradigms and the trajectory of future research on this topic.

### 4.1 Global research trends about methylation and glioma

The numerical growth of publications indicates the engagement of the research community with the topic of glioma methylation. From 2004 to the present, there has been an overall ascending trajectory in publication volume, punctuated by distinct chronological phases. Figure 2 shows that the initial period from 2004 to 2007 was characterized by a gradual increase in annual publications. Subsequently, a pronounced increase in the number of publications was observed from 2008 to 2018. After 2019, the publication volume witnessed a dramatic upsurge, which peaked in 2022, illustrating the heightened interest in this domain in recent years. With regard to national publication output, the United States was the leading contributor, followed by China, Germany, Italy, and Japan, respectively. Notably, the volume of publications from China closely rivaled that of the United States, and from the start of 2020, China secured the leading position in annual publication volume.

In this study, we used the metric of citation-to-publication ratio to appraise the quality of publications across various regions. Our findings unveiled that within the top 10 regions by publication volume, the United States secured the dominant position, with an impressive citation tally of 84,628 (Table 1). The citation-to-publication ratio of the United States, standing at 40.42, was notably high, signifying a high caliber of academic contributions. China, with a publication volume of 1,058, was the runner-up in terms of volume, yet its citation count of 23,152 placed it eighth. The citation-to-publication ratio was 21.88, suggesting a relative quality discrepancy. The recent upsurge in publication retractions by Chinese researchers is a concerning phenomenon that requires attention (Palla et al., 2020; Audisio et al., 2022; Kwee and Kwee, 2023). It is imperative to emphasize the importance of authentic research data and findings in the pursuit of scientific excellence.

On the other side, publications surge may be attributed to the efficacy of the glioma treatment regimen proposed by professor Stupp. This regimen involves the use of temozolomide for chemotherapy, a treatment strategy that targets the methylation status of the gene encoding O6-methylguanine-DNA methyltransferase (*MGMT*) (Hegi et al., 2004, 2005; Stupp et al., 2005). Furthermore, the advent of The Cancer Genome Atlas (TCGA) (Kim et al., 2010; Stegh et al., 2010) in 2006, a collaborative endeavor between the National Cancer Institute and the National Human Genome Research Institute, provided a genomic roadmap for 33 tumor types, with glioblastoma multiforme being a focal point in the initial 3 years of the study. This initiative has substantially underpinned glioma methylation research. The International Cancer Genome Consortium (Hudson et al., 2010), which was established in 2008, further augmented the omics data for malignant neoplasms. This spurred the progressive increase in research pertaining to tumor methylation (Jones et al., 2012). In China, the Chinese Glioma Genome Atlas (CGGA), initiated by professor Tao Jiang in 2012, has been instrumental in advancing the field of glioma omics (Bao et al., 2013, 2014; Wang and Jiang, 2013). The robust publication volume from the United States and mainland China is likely linked to the extensive utilization of the CGGA and TCGA databases. The advent of innovative analytical techniques has also drawn the attention of researchers to the field of glioma methylation, leading to a more nuanced and in-depth exploration of the field.

The collaboration network (Figure 5) illustrates a robust partnership between the United States and China, the two leading contributors. The United States has also formed close collaborations with Germany, the United Kingdom, Italy, and France, whereas China has shown a propensity for collaborations with Japan, Canada, and the Netherlands. The preeminence of the United States in this field is further accentuated by its substantial publication volume, frequent citations, and centrality score of 0.1. The institutional analysis revealed that 3,508 institutions contributed to glioma methylation research. Among the institutions in the top decile of publication volume, four were from the United States, and three were from Germany, while the others were from France, China, and Switzerland (Table 2, Figure 6). The Helmholtz Association achieved the most prolific publication record, with 253 articles accumulating 24,365 citations, averaging 96.30 citations per article. The German Cancer Research Center (DKFZ) was second, with 232 articles and 23,588 citations, averaging 101.67 citations per article. Ruprecht Karls University Heidelberg ranked third, with 196 articles and 27,033 citations, averaging 137.92 citations per article. The University of California System secured the fourth position, with 192 articles and 32,355 citations, averaging 168.52 citations per article. Our analysis indicated a preference for domestic collaboration among both national and international institutions. We advocate for a concerted effort to bolster cross-border institutional collaborations, which we believe would help to overcome academic silos and foster an environment that is conducive to unrestrained knowledge dissemination.

When deliberating on the most appropriate journal for their manuscripts, researchers frequently consult the body of literature already present within their field. In our analysis of pertinent journals, Journal of Neuro-Oncology was the leading platform, with the highest publication frequency. Among its most recent contributions, one study elucidated that the application of epigenetic editing technologies to modulate *MGMT* methylation status, or to regulate its methylation patterns in a rhythmic manner, can significantly impact the chemosensitivity of gliomas to temozolomide (Gonzalez-Aponte et al., 2024; Zapanta et al., 2024). These scholarly achievements not only serve as a benchmark for the dissemination of related research, but they also provide a theoretical framework for future translational application of *MGMT* methylation modulation in clinical glioma treatment.

The analysis of author contributions unveiled a compelling observation; despite the United States having the largest number of publications, the most prolific author did not reside within the research community of the United States. This suggests a robust and diverse research base within the United States, and the achievements were not overly centralized. Professor Michael Weller from Switzerland was the most prolific contributor, achieving seminal breakthroughs in the realm of glioma methylation as far back as 2002 (Watanabe et al., 2002; Baeza et al., 2003; Waltereit and Weller, 2003). Professor Tao Jiang of China was also distinguished among the leading authors, a status likely influenced by his role in establishing the CGGA. Moreover, professor Weller's work was recognized for its authoritative impact, as reflected in his ranking within the top quartile of citations. His research has progressively ventured into the domain of glioma immunotherapy, with an emphasis on the intricate immune microenvironment of glioma and the importance of devising targeted therapeutic strategies (Le Rhun et al., 2019; Hansch et al., 2023; Schmassmann et al., 2023).

### 4.2 Hot topics and frontiers in glioma and methylation research

One of the most efficacious strategies for discerning the focal points and future trajectories within a research field is the systematic dissection of keywords. Keywords allow the focal points of pertinent literature to be understood, and through the application of cluster analysis on frequently occurring keywords, a targeted examination of emergent terms. This approach clarifies the research hotspots and may predict the avenues along which future investigations are likely to proceed.

Upon analysis of the data provided in Table 5, “temozolomide” was identified as the predominant keyword, accompanied by a spectrum of interrelated keywords, including “survival,” “DNA methylation,” “*MGMT*,” “radiotherapy,” and “*MGMT* promoter methylation,” which were also highly ranked in terms of frequency.

Temozolomide is a cornerstone chemotherapeutic agent after surgical intervention for glioma, and its significance in the management of glioblastoma multiforme is particularly notable (Lee, 2016). Temozolomide exerts its tumor-inhibiting action by causing DNA methylation injury, predominantly targeting O6 guanine residues. This subsequently perturbs DNA replication and repair mechanisms. This disruption can initiate double-stranded DNA breaks and induce cellular apoptosis, thereby curtailing the proliferative and growth capacities of neoplastic cells (Strobel et al., 2019). Through its repair capabilities, *MGMT* plays a critical role in the cellular response to temozolomide-induced DNA damage. Therefore, elevated *MGMT* expression is commonly correlated with the diminished therapeutic efficacy of temozolomide. In stark contrast, the occurrence of *MGMT* promoter methylation is associated with diminished enzyme expression, which increases tumor susceptibility to temozolomide (Pegg and Byers, 1992; Kitange et al., 2009). In clinical practice, the methylation status of the *MGMT* promoter has been affirmed as a prognostic indicator, pivotal in forecasting the sensitivity of patients with glioma to temozolomide, as well as predicting their subsequent clinical outcomes (Melguizo et al., 2012; Mansouri et al., 2019).

In the evolving landscape of glioma research, there is a quest to identify therapeutic strategies that can overcome the resistance of glioma to temozolomide, which would have profound implications for improving patient outcomes. To date, the majority of these research efforts have been anchored within the foundational stages of experimental research (Xue et al., 2022; Nam et al., 2023; Zhou et al., 2023; Wang et al., 2024; Ye et al., 2024), yet they have managed to penetrate various pivotal biological mechanisms. These include the intricate processes of tumor energy metabolism, angiogenesis, the functional role of exosomes, lipid metabolism, and the complex interplay of epigenetic modifications. Innovative drug delivery systems are poised to provide a potential breakthrough in overcoming these therapeutic barriers (Wang et al., 2022).

The keyword burst analysis revealed that the period from 2019 to 2022 yielded a spectrum of keywords, which we systematically classified into two distinct categories. The first category was associated with methodologies, notably highlighting “deep learning” and “machine learning.” The second category pertained to research focus, which included the keywords “tumor microenvironment,” “immuno-microenvironment,” and “immune infiltration.” These keywords not only define the research hotspots of recent years but also underscore the evolving landscape of this field. The application of deep learning and machine learning techniques, with their robust analytical capabilities for complex datasets, has permeated various aspects of glioma management, ranging from diagnostics and differential diagnostics to the stratification and prognostic evaluation of glioma. Notably, the utilization of deep learning in conjunction with radiomics for the differential diagnosis of glioblastoma multiforme from other intracranial lesions (Nakagawa et al., 2018; Zhang et al., 2021) has demonstrated superior diagnostic efficacy. Furthermore, these analytical approaches have been effectively extended to the preoperative staging (Tian et al., 2018; Gutta et al., 2021; Li et al., 2022) and prognostic assessment (Kim et al., 2019; Feng et al., 2020; Metz et al., 2020) of glioma. Therefore, they have helped to enhance preoperative diagnostic precision, grading accuracy, genetic profile prediction, and survival evaluation. Collectively, these sophisticated analytical methodologies have led to significant advancements in the perioperative management of glioma, offering promising avenues for future research and therapeutic development.

The glioma microenvironment, a focal point within tumor immunology, is pivotal for elucidating the etiology, progression, invasive behavior, and mechanisms of chemoresistance. It also serves as a cornerstone for developing targeted therapeutic interventions. As shown in the published literature, the contributions of professor Michael Weller are intricately related to the immunological underpinnings of glioma (Naghavian et al., 2023). Professor Weller's team has spearheaded a therapeutic approach that synergizes the L19 antibody with the L19TNF fusion protein, coupled with the chemotherapeutic agent lomustine (CCNU). This regimen has demonstrated robust therapeutic efficacy in a murine model of orthotopic glioblastoma multiforme. The L19TNF fusion protein, with its specificity for the tumor neovasculature, in tandem with the alkylating properties of CCNU, has been shown to induce DNA damage within the tumor and facilitate tumor cell necrosis. The clinical efficacy of this synergistic therapeutic modality has been corroborated in a study registered under the identifier NCT04573192, where three of five patients with recurrent glioblastoma multiforme exhibited measurable objective remission (Look et al., 2023). In a subsequent investigation led by professor Weller (Hansch et al., 2023), the transmembrane protein CD317 was considered an innovative target for chimeric antigen receptor (CAR) T-cell therapeutics. CAR T-cells endowed with specificity for CD317 have demonstrated pronounced antitumor efficacy against glioma cells, thereby introducing a novel paradigm in the immunotherapeutic arsenal against glioblastoma multiforme. In parallel, the role of DNA methylation in the context of glioma immunotherapy is emerging as a subject of scrutiny. The establishment of a DNA methylation-based predictive model has been instrumental in the stratification of patients with low-grade glioma, and it holds potential as a biomarker for the prognostication of immunotherapeutic responses (Yang et al., 2022). Additionally, the methylation modification mediated by M6A has been identified to exert an influence over the immunological microenvironment of glioma, leading to the dysfunction of tumor-infiltrating T-cells and consequently impacting immunotherapy outcomes (Zhao et al., 2023). These findings underscore the multifaceted and dynamic nature of the glioma microenvironment and highlight the importance of continued exploration into the immunotherapeutic potential against this formidable disease entity.

Collectively, the body of work ranging from professor Weller's seminal studies to the growing potential of DNA methylation in the domain of immunotherapy exemplifies the intensive and innovative pursuit of therapeutic strategies for glioma. These insights have significantly enriched our understanding of the glioma microenvironment and have laid a robust scientific foundation and promising clinical horizon for the development of innovative targeted therapies. The collective impact of these advancements is far-reaching, offering a renewed sense of optimism for patients with glioma and leading toward a therapeutically diverse and efficacious future for glioma. These findings are instrumental in the ongoing quest to enhance the longevity and qualitative aspects of life for individuals battling this aggressive neurological malignancy.

## 5 Limitations

This study has several limitations that should be considered. First, our data analysis was limited to the WoSCC database, neglecting a broader spectrum of databases that could have been pertinent to our research. Second, aiming to refine the accuracy of our analysis, we elected to include only specific scholarly articles, namely original contributions and comprehensive reviews. Finally, despite the objectivity inherent in VOSviewer and CiteSpace, the subjective perspectives of the analysts may have exerted some influence on the interpretation of the findings. These considerations underscore the necessity for a more expansive and diverse approach in subsequent research endeavors.

## 6 Conclusions

Methylation is integral to the pathogenesis of glioma, with the epimutations of specific genes exerting a significant regulatory effect on the biological behavior of this type of tumor. Epigenetic editing techniques may be a novel strategic approach in clinical oncology, but although promising, this development presents unprecedented challenges. We exhaustively analyzed the literature on glioma methylation spanning almost two decades within the WoCSS database. Using bibliometric methodologies, we elucidated the origins of these publications, the contributing institutions, authorship, publishing journals, and keywords. This analysis provides an exhaustive overview of the current landscape and identifies the focal points of glioma methylation research. The evolution of novel analytical algorithms and the development of sophisticated research tools are anticipated to invigorate further exploration within this field. Particularly, the investigation into methylation-associated immunotherapies for glioma treatment is expected to be increasingly refined, facilitating an effective transition from basic scientific inquiry to clinical application.

## Data Availability

Publicly available datasets were analyzed in this study. This data can be found here: all datasets was in Supplementary material.

## References

[B1] Audisio K. Robinson N. B. Soletti G. J. Cancelli G. Dimagli A. Spadaccio C.. (2022). A survey of retractions in the cardiovascular literature. Int. J. Cardiol. 349, 109–114. 10.1016/j.ijcard.2021.12.021 34921899

[B2] Baeza N. Weller M. Yonekawa Y. Kleihues P. Ohgaki H. (2003). Pten methylation and expression in glioblastomas. Acta Neuropathol. 106, 479–485. 10.1007/s00401-003-0748-4 12904991

[B3] Bao Z. Zhang C. Yan W. Liu Y. Li M. Zhang W.. (2013). Bmp4, a strong better prognosis predictor, has a subtype preference and cell development association in gliomas. J. Transl. Med. 11:100. 10.1186/1479-5876-11-100 23590708 PMC3637580

[B4] Bao Z. S. Li M. Y. Wang J. Y. Zhang C. B. Wang H. J. Yan W.. (2014). Prognostic value of a nine-gene signature in glioma patients based on mrna expression profiling. CNS Neurosci. Ther. 20, 112–118. 10.1111/cns.12171 24279471 PMC6493176

[B5] Brat D. J. Verhaak R. G. W. Aldape K. D. Yung W. K. A. Salama S. R. Cooper L. A. D.. (2015). Comprehensive, integrative genomic analysis of diffuse lower-grade gliomas. N. Engl. J. Med. 372, 2481−2498. 10.1056/NEJMoa1402121 26061751 PMC4530011

[B6] Brennan C. W. Verhaak R. G. W. Mckenna A. Campos B. Noushmehr H. Salama S. R.. (2013). The somatic genomic landscape of glioblastoma. Cell 155, 462–477. 10.1016/j.cell.2013.09.034 24120142 PMC3910500

[B7] Capper D. Jones D. T. W. Sill M. Hovestadt V. Schrimpf D. Sturm D.. (2018a). Dna methylation-based classification of central nervous system tumours. Nature 555, 469–474. 10.1038/nature26000 29539639 PMC6093218

[B8] Capper D. Stichel D. Sahm F. Jones D. T. W. Schrimpf D. Sill M.. (2018b). Practical implementation of dna methylation and copy-number-based cns tumor. Acta Neuropathol. 136, 181–210. 10.1007/s00401-018-1879-y 29967940 PMC6060790

[B9] Ceccarelli M. Barthel F. P. Malta T. M. Sabedot T. S. Salama S. R. Murray B. A.. (2016). Molecular profiling reveals biologically discrete subsets and pathways of progression in diffuse glioma. Cell 164, 550–563. 10.1016/j.cell.2015.12.028 26824661 PMC4754110

[B10] Chen C. Leydesdorff L. (2014). Patterns of connections and movements in dual-map overlays: a new method of publication portfolio analysis. J. Assoc. Inform. Sci. Technol. 65, 334–351. 10.1002/asi.22968

[B11] Du J. Ji H. Ma S. Jin J. Mi S. Hou K.. (2021). M6a regulator-mediated methylation modification patterns and characteristics of. Brief. Bioinform. 22:bbab013. 10.1093/bib/bbab013 33594424

[B12] Feng X. Tustison N. J. Patel S. H. Meyer C. H. (2020). Brain tumor segmentation using an ensemble of 3d u-nets and overall survival prediction using radiomic features. Front. Comput. Neurosci. 14:25. 10.3389/fncom.2020.00025 32322196 PMC7158872

[B13] Gonzalez-Aponte M. F. Damato A. R. Trebucq L. L. Simon T. Cardenas-Garcia S. P. Cho K.. (2024). Circadian regulation of MGMT expression and promoter methylation underlies daily rhythms in tmz sensitivity in glioblastoma. J. Neurooncol. 166, 419–430. 10.1007/s11060-023-04535-9 38277015 PMC11301575

[B14] Gutta S. Acharya J. Shiroishi M. S. Hwang D. Nayak K. S. (2021). Improved glioma grading using deep convolutional neural networks. AJNR Am. J. Neuroradiol. 42, 233–239. 10.3174/ajnr.A6882 33303522 PMC7872170

[B15] Hansch L. Peipp M. Mastall M. Villars D. Myburgh R. Silginer M.. (2023). Chimeric antigen receptor t cell-based targeting of cd317 as a novel immunotherapeutic strategy against glioblastoma. Neuro-Oncology 25, 2001–2014. 10.1093/neuonc/noad108 37335916 PMC10628943

[B16] Hegi M. E. Diserens A. Gorlia T. Hamou M. de Tribolet N. Weller M.. (2005). MGMT gene silencing and benefit from temozolomide in glioblastoma. N Engl J Med. 352, 997–1003. 10.1056/NEJMoa043331 15758010

[B17] Hegi M. E. Diserens A. C. Godard S. Dietrich P. Y. Regli L. Ostermann S.. (2004). Clinical trial substantiates the predictive value of o-6-methylguanine-dna methyltransferase promoter methylation in glioblastoma patients treated with temozolomide. Clin. Cancer Res. 10, 1871–1874. 10.1158/1078-0432.CCR-03-0384 15041700

[B18] Hudson T. J. Anderson W. Artez A. Barker A. D. Bell C. Bernabe R. R.. (2010). International network of cancer genome projects. Nature 464, 993–998. 10.1038/nature08987 20393554 PMC2902243

[B19] Jiang C. Hu Y. Wang S. Chen C. (2023). Emerging trends in dna and rna methylation modifications in type 2 diabetes mellitus: a bibliometric and visual analysis from 1992 to 2022. Front. Endocrinol. 14:1145067. 10.3389/fendo.2023.1145067 37201099 PMC10187586

[B20] Jones D. T. Jager N. Kool M. Zichner T. Hutter B. Sultan M.. (2012). Dissecting the genomic complexity underlying medulloblastoma. Nature 488, 100–105. 10.1038/nature11284 22832583 PMC3662966

[B21] Kim H. Huang W. Jiang X. Pennicooke B. Park P. J. Johnson M. D. (2010). Integrative genome analysis reveals an oncomir/oncogene cluster regulating glioblastoma survivorship. Proc. Natl. Acad. Sci. U. S. A. 107, 2183–2188. 10.1073/pnas.0909896107 20080666 PMC2836668

[B22] Kim J. Y. Park J. E. Jo Y. Shim W. H. Nam S. J. Kim J. H.. (2019). Incorporating diffusion- and perfusion-weighted mri into a radiomics model improves diagnostic performance for pseudoprogression in glioblastoma patients. Neuro-Oncol. 21, 404–414. 10.1093/neuonc/noy133 30107606 PMC6380413

[B23] Kitange G. J. Carlson B. L. Mladek A. C. Decker P. A. Schroeder M. A. Wu W.. (2009). Evaluation of MGMT promoter methylation status and correlation with temozolomide response in orthotopic glioblastoma xenograft model. J. Neurooncol. 92, 23–31. 10.1007/s11060-008-9737-8 19011762 PMC2790867

[B24] Kwee R. M. Kwee T. C. (2023). Retracted publications in medical imaging literature: an analysis using the retraction watch database. Acad. Radiol. 30, 1148–1152. 10.1016/j.acra.2022.06.025 35977877

[B25] Le Rhun E. Preusser M. Roth P. Reardon D. A. van den Bent M. Wen P.. (2019). Molecular targeted therapy of glioblastoma. Cancer Treat. Rev. 80:101896. 10.1016/j.ctrv.2019.101896 31541850

[B26] Lee S. Y. (2016). Temozolomide resistance in glioblastoma multiforme. Genes Dis. 3, 198–210. 10.1016/j.gendis.2016.04.007 30258889 PMC6150109

[B27] Li Y. Wei D. Liu X. Fan X. Wang K. Li S.. (2022). Molecular subtyping of diffuse gliomas using magnetic resonance imaging: comparison and correlation between radiomics and deep learning. Eur. Radiol. 32, 747–758. 10.1007/s00330-021-08237-6 34417848

[B28] Look T. Puca E. Bühler M. Kirschenbaum D. De Luca R. Stucchi R.. (2023). Targeted delivery of tumor necrosis factor in combination with ccnu induces a T cell-dependent regression of glioblastoma. Sci. Transl. Med. 15:eadf2281. 10.1126/scitranslmed.adf2281 37224228

[B29] Louis D. N. Perry A. Reifenberger G. von Deimling A. Figarella-Branger D. Cavenee W. K.. (2016). The 2016 world health organization classification of tumors of the central nervous system: a summary. Acta Neuropathol. 131, 803–820. 10.1007/s00401-016-1545-1 27157931

[B30] Louis D. N. Perry A. Wesseling P. Brat D. J. Cree I. A. Figarella-Branger D.. (2021). The 2021 who classification of tumors of the central nervous system: a summary. Neuro-Oncol. 23, 1231–1251. 10.1093/neuonc/noab106 34185076 PMC8328013

[B31] Mansouri A. Hachem L. D. Mansouri S. Nassiri F. Laperriere N. J. Xia D.. (2019). MGMT promoter methylation status testing to guide therapy for glioblastoma: refining the approach based on emerging evidence and current challenges. Neuro-Oncol. 21, 167–178. 10.1093/neuonc/noy132 30189035 PMC6374759

[B32] Mao M. Chu Q. Lou Y. Lv P. Wang L. (2022). Rna n1-methyladenosine regulator-mediated methylation modification patterns and. Front. Immunol. 13:948630. 10.3389/fimmu.2022.948630 35936006 PMC9354098

[B33] Mao Y. Zhao K. Chen N. Fu Q. Zhou Y. Kong C.. (2023). A 2-decade bibliometric analysis of epigenetics of cardiovascular disease: from past to present. Clin. Epigenetics 15:184. 10.1186/s13148-023-01603-9 38007493 PMC10676610

[B34] Melguizo C. Prados J. Gonzalez B. Ortiz R. Concha A. Alvarez P. J.. (2012). MGMT promoter methylation status and MGMT and CD133 immunohistochemical expression as prognostic markers in glioblastoma patients treated with temozolomide plus radiotherapy. J. Transl. Med. 10:250. 10.1186/1479-5876-10-250 23245659 PMC3551841

[B35] Metz M. C. Molina-Romero M. Lipkova J. Gempt J. Liesche-Starnecker F. Eichinger P.. (2020). Predicting glioblastoma recurrence from preoperative mr scans using fractional-anisotropy maps with free-water suppression. Cancers 12:728. 10.3390/cancers12030728 32204544 PMC7140058

[B36] Naghavian R. Faigle W. Oldrati P. Wang J. Toussaint N. C. Qiu Y.. (2023). Microbial peptides activate tumour-infiltrating lymphocytes in glioblastoma. Nature 617, 807–817. 10.1038/s41586-023-06081-w 37198490 PMC10208956

[B37] Nakagawa M. Nakaura T. Namimoto T. Kitajima M. Uetani H. Tateishi M.. (2018). Machine learning based on multi-parametric magnetic resonance imaging to differentiate glioblastoma multiforme from primary cerebral nervous system lymphoma. Eur. J. Radiol. 108, 147–154. 10.1016/j.ejrad.2018.09.017 30396648

[B38] Nam Y. Koo H. Yang Y. Shin S. Zhu Z. Kim D.. (2023). Pharmacogenomic profiling reveals molecular features of chemotherapy resistance in idh wild-type primary glioblastoma. Genome Med. 15:16. 10.1186/s13073-023-01165-8 36915208 PMC10010007

[B39] Ozair A. Bhat V. Alisch R. S. Khosla A. A. Kotecha R. R. Odia Y.. (2023). Dna methylation and histone modification in low-grade gliomas: current. Cancers 15:1342. 10.3390/cancers15041342 36831683 PMC9954183

[B40] Palla I. A. Singson M. Thiyagarajan S. (2020). A comparative analysis of retracted papers in health sciences from china and india. Account. Res. 27, 401–416. 10.1080/08989621.2020.1754804 32279538

[B41] Pan T. Wu F. Li L. Wu S. Zhou F. Zhang P.. (2021). The role m(6)a rna methylation is cns development and glioma pathogenesis. Mol. Brain 14:119. 10.1186/s13041-021-00831-5 34281602 PMC8290532

[B42] Pegg A. E. Byers T. L. (1992). Repair of dna containing o6-alkylguanine. FASEB. J. 6, 2302–2310. 10.1096/fasebj.6.6.1544541 1544541

[B43] Romani M. Pistillo M. P. Banelli B. (2018). Epigenetic targeting of glioblastoma. Front. Oncol. 8:448. 10.3389/fonc.2018.00448 30386738 PMC6198064

[B44] Schmassmann P. Roux J. Buck A. Tatari N. Hogan S. Wang J.. (2023). Targeting the siglec-sialic acid axis promotes antitumor immune responses in preclinical models of glioblastoma. Sci. Transl. Med. 15:eadf5302. 10.1126/scitranslmed.adf5302 37467314

[B45] Sharma M. Barravecchia I. Magnuson B. Ferris S. F. Apfelbaum A. Mbah N. E.. (2023). Histone h3 k27m-mediated regulation of cancer cell stemness and differentiation. Neoplasia 44:100931. 10.1016/j.neo.2023.100931 37647805 PMC10474232

[B46] Stegh A. H. Brennan C. Mahoney J. A. Forloney K. L. Jenq H. T. Luciano J. P.. (2010). Glioma oncoprotein bcl2l12 inhibits the p53 tumor suppressor. Genes. Dev. 24, 2194–2204. 10.1101/gad.1924710 20837658 PMC2947771

[B47] Strobel H. Baisch T. Fitzel R. Schilberg K. Siegelin M. D. Karpel-Massler G.. (2019). Temozolomide and other alkylating agents in glioblastoma therapy. Biomedicines 7:69. 10.3390/biomedicines7030069 31505812 PMC6783999

[B48] Stupp R. Hegi M. E. Mason W. P. van den Bent M. J. Taphoorn M. J. B. Janzer R. C.. (2009). Effects of radiotherapy with concomitant and adjuvant temozolomide versus radiotherapy alone on survival in glioblastoma in a randomised phase iii study: 5-year analysis of the eortc-ncic trial. Lancet. Oncol. 10, 459–466. 10.1016/S1470-2045(09)70025-7 19269895

[B49] Stupp R. van den Bent M. J. Hegi M. E. (2005). Optimal role of temozolomide in the treatment of malignant gliomas. Curr. Neurol. Neurosci. Rep. 5, 198–206. 10.1007/s11910-005-0047-7 15865885

[B50] Tian Q. Yan L. F. Zhang X. Zhang X. Hu Y. C. Han Y.. (2018). Radiomics strategy for glioma grading using texture features from multiparametric mri. J. Magn. Reson. Imaging 48, 1518–1528. 10.1002/jmri.26010 29573085

[B51] van Eck N. J. Waltman L. (2010). Software survey: vosviewer, a computer program for bibliometric mapping. Scientometrics 84, 523–538. 10.1007/s11192-009-0146-3 20585380 PMC2883932

[B52] Verhaak R. G. W. Hoadley K. A. Purdom E. Wang V. Qi Y. Wilkerson M. D.. (2010). Integrated genomic analysis identifies clinically relevant subtypes of glioblastoma characterized by abnormalities in PDGFRA, IDH1, EGFR, and NF1. Cancer Cell 17, 98–110. 10.1016/j.ccr.2009.12.020 20129251 PMC2818769

[B53] Waltereit R. Weller M. (2003). Signaling from camp/pka to mapk and synaptic plasticity. Mol. Neurobiol. 27, 99–106. 10.1385/MN:27:1:99 12668903

[B54] Wang H. Jiang C. (2013). Rab38 confers a poor prognosis, associated with malignant progression and subtype preference in glioma. Oncol. Rep. 30, 2350–2356. 10.3892/or.2013.2730 24026199

[B55] Wang M. Xia D. Xu D. Yin Y. Xu F. Zhang B.. (2024). Neovascularization directed by CAVIN1/CCBE1/VEGFC confers tmz-resistance in glioblastoma. Cancer Lett. 582, 216593. 10.1016/j.canlet.2023.216593 38092144

[B56] Wang R. Liang Q. Zhang X. Di Z. Wang X. Di L. (2022). Tumor-derived exosomes reversing TMZ resistance by synergistic drug delivery for glioma-targeting treatment. Colloid Surf. B-Biointerf. 215:112505. 10.1016/j.colsurfb.2022.112505 35487070

[B57] Watanabe T. Huang H. Nakamura M. Wischhusen J. Weller M. Kleihues P.. (2002). Methylation of the P73 gene in gliomas. Acta Neuropathol. 104, 357–362. 10.1007/s00401-002-0549-1 12200621

[B58] Wick W. Weller M. van den Bent M. Sanson M. Weiler M. von Deimling A.. (2014). MGMT testing–the challenges for biomarker-based glioma treatment. Nature reviews. Neurology 10, 372–385. 10.1038/nrneurol.2014.100 24912512

[B59] Xue Y. Y. Lu Y. Y. Sun G. Q. Fang F. Ji Y. Q. Tang H. F.. (2022). Cn-3 increases tmz sensitivity and induces ros-dependent apoptosis and autophagy in tmz-resistance glioblastoma. J. Biochem. Mol. Toxicol. 36:e22973. 10.1002/jbt.22973 34967073

[B60] Yan H. Parsons D. W. Jin G. Mclendon R. Rasheed B. A. Yuan W.. (2009). IDH1 and IDH2 mutations in gliomas. N. Engl. J. Med. 360, 765–773. 10.1056/NEJMoa0808710 19228619 PMC2820383

[B61] Yang G. Shan D. Zhao R. Li G. (2022). Metabolism-associated dna methylation signature stratifies lower-grade glioma patients and predicts response to immunotherapy. Front. Cell. Dev. Biol. 10:902298. 10.3389/fcell.2022.902298 35784470 PMC9240391

[B62] Ye L. Gu L. Wang Y. Xing H. Li P. Guo X.. (2024). Identification of tmz resistance-associated histone post-translational modifications in glioblastoma using multi-omics data. CNS Neurosci. Ther. 30:e14649. 10.1111/cns.14649 38448295 PMC10917648

[B63] Zapanta R. S. Li T. Pianka S. T. Prins T. J. Eldred B. Kevan B. M.. (2024). Dcas9/crispr-based methylation of o-6-methylguanine-dna methyltransferase enhances chemosensitivity to temozolomide in malignant glioma. J. Neurooncol. 166, 129–142. 10.1007/s11060-023-04531-z 38224404 PMC10824881

[B64] Zhang Y. Liang K. He J. Ma H. Chen H. Zheng F.. (2021). Deep learning with data enhancement for the differentiation of solitary and multiple cerebral glioblastoma, lymphoma, and tumefactive demyelinating lesion. Front. Oncol. 11:665891. 10.3389/fonc.2021.665891 34490082 PMC8416477

[B65] Zhang Y. Zhu J. (2019). Ten genes associated with MGMT promoter methylation predict the prognosis of. Oncol. Rep. 41, 908–916. 10.3892/or.2018.6903 30535433 PMC6313003

[B66] Zhao B. Xiang Z. Wu B. Zhang X. Feng N. Wei Y.. (2023). Use of novel m6a regulator-mediated methylation modification patterns in distinct tumor microenvironment profiles to identify and predict glioma prognosis and progression, T-cell dysfunction, and clinical response to ici immunotherapy. Curr. Pharm. Des. 29, 60–78. 10.2174/1381612829666221207112438 36503445

[B67] Zhou D. Wan Y. Xie D. Wang Y. Wei J. Yan Q.. (2015). Dnmt1 mediates chemosensitivity by reducing methylation of mirna-20a promoter in. Experim. Mol. Med. 47:e182. 10.1038/emm.2015.57 26337869 PMC4650929

[B68] Zhou J. Tong F. Zhao J. Cui X. Wang Y. Wang G.. (2023). Identification of the E2F1-RAD51AP1 axis as a key factor in MGMT-methylated GBM TMZ resistance. Cancer Biol. Med. 20, 385–400. 10.20892/j.issn.2095-3941.2023.0011 37283490 PMC10246439

